# Cadmium, Iron Deficiency Anemia and Hypophosphatemic Osteomalacia Due to Intravenous Iron Supplementation

**DOI:** 10.3390/biomedicines14020292

**Published:** 2026-01-28

**Authors:** Aleksandar Cirovic, Petar Milovanovic, Soisungwan Satarug

**Affiliations:** 1 Center of Bone Biology, Faculty of Medicine, Institute of Anatomy, University of Belgrade, 11000 Belgrade, Serbia; aleksandar.cirovic@med.bg.ac.rs; 2 Centre for Kidney Disease Research, Translational Research Institute, The University of Queensland, Woolloongabba, Brisbane, QLD 4102, Australia

**Keywords:** anemia, bilirubin, bone fragility, cadmium, fibroblast growth factor 23, heme oxygenase-1, iron deficiency, osteomalacia, zinc

## Abstract

Cadmium (Cd) is a ubiquitous environmental pollutant that enters the circulation from the lungs and gastrointestinal tract. For most people, staple foods form the main route of Cd exposure. Current evidence suggests that Cd may increase the prevalence of iron deficiency and anemia in environmentally exposed people. Concerningly, intravenous iron administration to treat iron deficiency anemia has resulted in adverse bone outcomes at a higher-than-expected frequency, for which reasons remain unclear. The bone-derived hormone fibroblast growth factor 23 (FGF23), the regulator of vitamin D and phosphate homeostasis, has been speculated to be implicated, given that anemia, iron deficiency and inflammatory conditions are all known to increase FGF23 expression levels in osteoblasts. Additionally, early studies have demonstrated that Cd increases FGF23 expression by osteoblast-like cells and suppresses FGF23 cleavage, leading to an abrupt rise in serum FGF23, which, in turn, mediates an effect of Cd on tubular phosphate reabsorption. In this review, experimental breakthrough studies showing Cd-induced iron deficiency and a reduction in iron absorption by Cd are summarized, together with intestinal absorption of Cd and an increment in Cd uptake and Cd body burden in those with low body iron stores. Potential contributions of Cd, anemia and iron deficiency in the context of hypophosphatemic osteomalacia development after intravenous iron supplementation are discussed. The molecular basis of Cd-induced ferroptosis in pathogenesis of osteoporosis, emphasizing heme oxygenase-1 (HO-1)/bilirubin axis and zinc deficiency, is presented.

## 1. Introduction

Iron deficiency anemia (IDA) is defined based on levels of blood hemoglobin and indicators of body iron content, like serum ferritin, free erythrocyte protoporphyrin and the transferrin saturation index [1,2,3,4,5]. Ferritin is an iron storage protein; as such, a ferritin concentration in the serum below 20 and 30 µg/L is indicative of depleted and low body iron stores, respectively [2,6]. However, because of the rising serum ferritin levels in chronic inflammatory conditions, soluble transferrin receptors (sTfR) and the sTfR–ferritin index have been employed for accurate IDA assessment [2,6].

Based on blood hemoglobin levels below 12.0 g/dL in women and 13.0 g/dL in men, the 2021 global prevalence of anemia across all ages was 24.3% [7,8]. The predominant cause of anemia is iron deficiency, followed by hemoglobinopathies, hemolysis and chronic disease. IDA is a significant problem, especially among children (1–4 years), women of childbearing age, pregnant women [9,10,11] and people with chronic diseases, including chronic kidney disease (CKD) [6,12,13]. Moreover, IDA is a known risk factor for osteoporosis [14,15], while a high risk of bone fracture has been linked to low blood hemoglobin levels in men [16]. In a comprehensive review, Lichtler and Cowley presented evidence connecting the prevalence of IDA and anemia with environmental exposure to cadmium (Cd), lead (Pb), fluoride (F) and indoor and ambient air pollution [17].

Oral and intravenous iron supplementation is in current use for IDA treatment and management [18,19,20]. Intravenous iron administration is used to bypass the gastrointestinal tract because of the low absorption rate of oral iron and its side effects. Iron infusion therapy increased hemoglobin concentrations more efficiently than oral iron in CKD patients and resulted in higher hemoglobin and ferritin levels in pregnant women, compared with oral iron [10,11,12,13].

Concerningly, hypophosphatemic osteomalacia occurred in a particularly high proportion (50%) of patients who received intravenous iron therapy using a ferric carboxymaltose preparation [19,20]. Fibroblast growth factor 23 (FGF23), a bone-derived hormone, has been implicated; specifically, ferric carboxymaltose may inhibit the cleavage of FGF23, leading to an abrupt rise in active, intact FGF23 (iFGF23) [19,20]. Notably, iron deficiency per se can affect both synthesis and degradation of FGF23, from which iFGF23 and cleaved C-terminal FGF23 [(Cter)-FGF23] peptides are generated [21], and a Cter-FGF23 fragment has been linked to iron homeostasis, independent of the effect of iFGF23 on renal phosphate reabsorption [22]. Moreover, other intravenous iron formulations have also resulted in hypophosphatemic osteomalacia, albeit at low frequencies (4–5%).

Hypophosphatemic osteomalacia due to high dietary Cd exposure (>100 µg/day), experienced by itai-itai disease patients, is now rare; however, the evidence connecting iron deficiency with environmental Cd exposure [17], together with hypophosphatemia osteomalacia in iron infusion therapy [19,20], has prompted us to publish the present work. Effects of Cd on intestinal absorption of iron, leading to iron deficiency, are highlighted, along with effects of IDA on Cd absorption and the body burden of Cd. We discuss the potential role of Cd in the context of adverse bone outcomes of iron infusion therapy. Additionally, we provide insights into effects of Cd on cellular stress response mechanisms involving heme oxygenase-1 (HO-1)/bilirubin axis. The two-hit hypothesis of Cd-induced cytotoxicity is presented.

## 2. Absorption and Accumulation of Cd in the Human Body

In this section, the intestinal absorption of Cd is discussed, along with the use of blood Cd and urinary Cd as indicators of exposure to the metal. The effects of Cd on iron assimilation and reduced cellular iron uptake, revealed in recent long-term feeding trials, are highlighted. Special emphasis is given to increased Cd absorption and Cd body burden in those with low body iron status.

### 2.1. Cd Exposure Route and Bone Outcome

Cd is present as a contaminant in virtually all food types, especially staple foods; inevitably, normal diets have become a common route of exposure [23,24,25]. Additional Cd exposure routes are tobacco smoke and airborne particle pollution, a concern that exists especially among urban populations [26,27,28,29]. Exposure to a Cd dose between 10 and 15 µg/day may increase the risk of osteoporosis [30,31,32].

In high doses, daily exposure to Cd at 100 µg or lifetime exposure to 1 g can cause itai-itai disease [33,34], where osteomalacia was observed in the presence of markedly reduced proximal tubular reabsorption of various filtered substances, phosphate included [35,36,37,38]. These manifestations of severe Cd poisoning were replicated using ovariectomized cynomolgus monkeys [39]. In a study using osteoblast-like cells, Kido et al. found that Cd increased expression of FGF23, the regulator of vitamin D and phosphate homeostasis [40]. In a study by Aranami et al., a reduction in kidney tubular phosphate reabsorption in Cd-intoxicated mice was found to be mediated by FGF23 [41].

### 2.2. Intestinal Absorption of Cd

**Figure 1:**
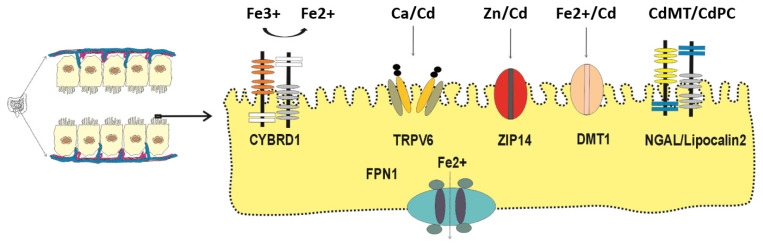
Specialized transport proteins and receptors expressed by enterocytes. The metal transporters involved in Cd absorption are those for calcium (TRPV6), zinc (ZIP14), iron (DMT1). Cd-bound metallothionine (CdMT) and Cd-bound phytochelatin (CdPC) are absorbed by transcytosis and endocytosis mediated by NGAL/lipocalin 2 receptor. Abbreviations: CYBRD1, cytochrome b reductase 1; FPN1, ferroportin1; TRPV6, transient receptor potential vanilloid6; ZIP14, Zrt- and Irt-related protein 14; DMT1, divalent metal transporter 1; MT, metallothionine; PC, phytochelatin; NGAL, neutrophil gelatinase-associated lipocalin.

All living organisms can neither generate nor destroy any metal; consequently, specialized metal transport proteins and pathways have evolved to acquire all essential metals, iron, zinc, manganese, calcium and cobalt, from exogenous sources (diet) [42,43,44]. Even though Cd has no physiological role or nutritional value, its electronegativity and ionic radius are close to those of zinc, calcium and iron; consequently, it is absorbed by enterocytes through the transport mechanisms and pathways for essential metals (Figure 1).

Examples of metal transport proteins responsible for absorption of Cd in an ionic form (Cd^2+^) are those for calcium (TRPV6), zinc (ZIP14) and iron (DMT1) [45,46,47,48,49,50]. Cd in complexes with metallothionein (MT) and the plant metal-binding ligand phytochelatin (PC) are absorbed through transcytosis [51] and endocytosis mediated by human neutrophil gelatinase-associated lipocalin (hNGAL)/lipocalin 2 receptor [52,53].

The rate of individual metal assimilation is regulated by the specificity and the levels of individuals transporters expressed by enterocytes. Such regulatory modes are pivotal to prevent metal deficiency as well as metal overload. Two metal efflux transporters have been found to display an absolute specificity; ferroportin1 (FNP1) serves as an exit route for iron (Fe^2+^) only [54,55], whereas ZnT1 is for the extrusion of Zn (2+) only [56]. These suggest that Cd is retained within cells due to the absence of exit route, leading to a long residence time of Cd in cells.

The transport protein being for iron or zinc only also means that the absorption rate of Cd exceeds that of iron or zinc, given that Cd enters the enterocytes through multiple routes. At any given time, the whole-blood Cd level is indicative of recent exposure because the average lifespan of erythrocytes is 120 days. The biological half-life of blood Cd ranges between 75 and 128 days [57]. The half-life of Cd in the body varies from 7.4 to 30 years; the lower the bodily burden, the longer the half-life of Cd [58,59,60].

### 2.3. Urinary Cd Is Indicative of Body Burden and Toxicity at the Present Time

Acquired Cd accumulates mostly within the kidney tubular cells, where its levels increase up to the age of 50 years but decline thereafter due to its release into the urine as injured tubular cells die for any reason [61]. Thus, urinary Cd reflects its nephrotoxicity at the present time [61]. A study of environmentally exposed Chinese subjects aged 2.8 to 86.8 years (*n* = 1235) showed that Cd excretion levels increased with age, peaking at 50 years in non-smoking women and 60 years in non-smoking men [62].

Based on a direct relationship between urinary Cd and its accumulation in the kidney cortex, urinary Cd is an indicator of the body burden of the metal [63,64,65,66]. Using data from kidney transplant donors, urinary Cd of 0.42 μg/g cr corresponded to kidney Cd of 25 μg/g wet tissue weight [65]; urinary Cd 0.34 μg/g cr in women corresponded to kidney Cd of 17.1 μg/g and urinary Cd of 0.23 μg/g cr in men corresponded to kidney Cd of 12.5 μg/g [66].

### 2.4. Iron Deficiency Induced by Cd: Breakthrough Studies

Using a long-term feeding strategy, Tokumoto et al. have shown, for the first time, an effect of Cd on iron absorption, leading to iron deficiency in Cd toxicity targets [67]. A significant reduction in hepatic iron content was observed in groups of female C57BL/6J mice given a diet containing 300 ppm Cd for 12, 15, 19 and 21 months [67]. Such a decrease in hepatic iron content was attributable to Cd-induced suppression of duodenal expression of the HCP1 and Cybrd1 genes, encoding the influx transporters for heme iron and non-heme iron, respectively. As Figure 1 presents, CYBRD1 is required to reduce Fe^3+^ to its absorbable form, Fe^2+^. Interestingly, Cd did not seem to influence the duodenal expression of iron efflux transporters [67]. The conditions may favor Cd absorption. In summary, Cd reduced duodenal absorption of both heme iron and non-heme iron, leading to a decrease in hepatic iron storage (ferritin), indicative of low body iron stores.

In another breakthrough study, where Sprague Dawley male rats were given Cd in drinking water at 0, 50, or 75 mg/L CdCl_2_ for 1 and 6 months, Zhang et al. observed a decrease in iron content in the proximal tubular cells of the kidneys [68]. They found, like the study by Tokumoto et al., that Cd affected the duodenal expression of the genes encoding specialized transport proteins for metals. Specifically, Cd suppressed the expression of SLC11A2 and SLC40A1 genes, encoding DMT1 and FPN1. Additionally, Cd lowered the expression levels of various metal transporters by the kidney tubular cells, which included SLC11A2 (DMT1), CUBN (cubilin), LRP2 (megalin), SLC39A14 (ZIP14) and SLC39A8 (ZIP8). Thus, long-term Cd exposure may induce an iron-insufficient state in tubular cells and interfere with protein endocytosis mediated by cubilin/megalin through decreasing the duodenal absorption, systemic transport and uptake of iron by tubular cells.

The above findings lend support to a connection between low environmental exposure to Cd and a high prevalence of iron deficiency, especially in vulnerable subpopulation groups [17]. They also prompted us to explore the potential impact of Cd on undesirable bone outcomes in intravenous iron supplementation, through inducing iron deficiency (Section 3).

## 3. Undesirable Bone Outcomes in Iron Infusion Therapy

Speculatively, hypophosphatemic osteomalacia was linked to the use of a ferric carboxymaltose preparation. Nonetheless, such bone outcomes also occurred with other iron formulations [19,20], which may indicate potential involvement of different pathogenic factors. Recent research studies show that inflammation, anemia and dysregulated iron homeostasis (overload/deficiency) can impact FGF23 synthesis and its cleavage to iFGF23 and Cter-FGF23 [21,22]. Moreover, the Cter-FGF23 fragment may have a biological role distinct from iFGF-23 [21,22].

To reveal similarities/differences in pathological features, we review reported cases of bone complications following iron infusion therapy. Also, we review a proven case of hypophosphatemic osteomalacia in an Indian jewelry male worker exposed to a high dose of Cd in fumes [69]. His blood Cd level was 6 times higher than the occupational exposure limit of 5 µg/L, while his 24 h urinary Cd excretion was 51 µg [69].

### 3.1. Hypophosphatemic Osteomalacia

Samões et al. reported a case of a 70-year-old man who had Rendu–Osler–Weber disease and developed hypophosphatemic osteomalacia as a complication of frequent infusions of ferric carboxymaltose for more than 10 years [70]. Vilaca et al. have published a systematic review, in which the authors included 28 case reports (30 patients, aged 28–80 years) of osteomalacia developed as a complication of intravenous iron infusions [71]. Initially, all 30 individuals had IDA from gastrointestinal diseases or gynecological bleeding. These subjects underwent treatment with repeated intravenous iron infusions using various iron preparations (saccharated ferric oxide, iron polymaltose and ferric carboxymaltose), which led to osteomalacia marked by musculoskeletal pain, fractures and pseudofractures. Most cases had rising plasma iFGF23 levels; nonetheless, Cter-FGF23 levels were also elevated in some cases, along with hypophosphatemia and increased plasma FGF23 levels. The clinical picture involved bone pain and onset of fractures. Additionally, some of the included subjects had comorbidities such as Crohn’s disease, or were treated with glucocorticoids [72], both of which may significantly influence bone quality [73,74].

Establishing a link between intravenous iron supplementation and the onset of osteomalacia is a challenging task. Namely, the majority or all of these individuals had IDA prior to iron infusions, and IDA itself may damage bones [75,76,77,78]. Thus, the effects of IDA and iron infusion were indistinguishable. Moreover, individuals with IDA may have had elevated blood Cd concentrations [79,80,81], which are toxic to bones [82]. It was not possible to determine whether osteomalacia is a consequence of IDA, Cd toxicity, or a transient iron overload.

A case report on Cd-induced hypophosphatemic osteomalacia was recently published. The authors reported a case of a man in his 40s who complained of lower-back pain. Using X-ray imaging, pseudofractures were observed [83]. His blood Cd level of 30 µg/L was sixfold above an occupational exposure limit of 5.0 µg/L. He also had hypochromic microcytic anemia and elevated Cter-FGF23 and was ultimately diagnosed with hypophosphatemic osteomalacia due to severe Cd toxicity [83].

A comparative overview of the main characteristics of cases of reported iron infusion-mediated osteomalacia and cases of Cd-mediated osteomalacia shows that the two conditions share all major characteristics, namely the presence of IDA, increased plasma FGF23 levels, hypophosphatemia and bone pain. Unfortunately, Cd exposure levels were not measured in the cases where iron was found to be “guilty” for the onset of hypophosphatemic osteomalacia; measuring Cd exposure levels in those cases would be helpful to clarify whether Cd contributed to or caused osteomalacia.

Changing therapy from intravenous to oral administration of iron brings improvement in those individuals treated with intravenous iron supplementation [71]. This improvement can be easily explained since Cd and iron share the same intestinal metal transporters and pathways (Section 2.2). DMT1, ZIP8 and ZIP14 metal transporters are expressed in the duodenum [84], and they are likely responsible for assimilation of iron, zinc and Cd [85,86]. This further suggests that iron and Cd would compete for the same metal transport proteins; consequently, less Cd would enter the circulation in response to oral iron supplementation.

**Figure 2:**
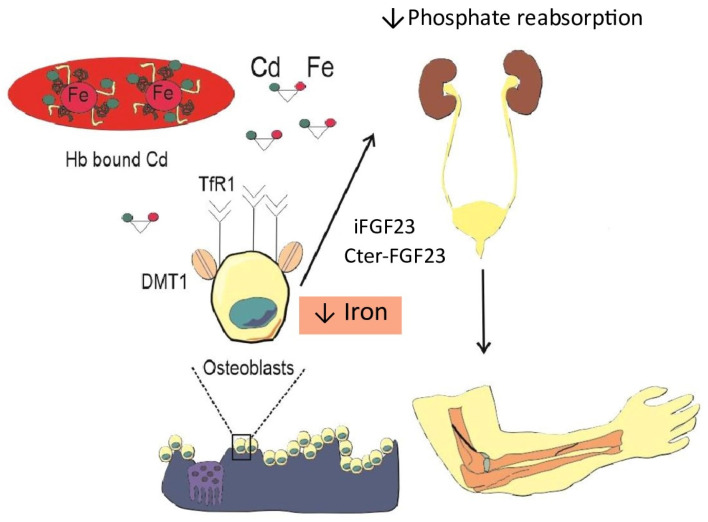
Cd-induced iron deficiency and hypophosphatemia. Through the transferrin receptor 1 (TfR1), Cd readily enters the osteoblasts. In response, osteoblasts secrete fibroblast growth factor 23 (FGF23), which reduces phosphate reabsorption by kidney proximal tubular cells. Iron deficiency due to Cd may influence the cleavage of FGF23 to iFGF23 and Cter-FGF23.

Interestingly, although it was speculated that plasma FGF23 levels would drop after the cessation of intravenous iron supplementation, Klein et al. reported that FGF23 remained elevated for months after the last infusion [87]. This may indicate involvement of factors influencing FGF23 synthesis and cleavage. This should not suggest that hypophosphatemic osteomalacia is the consequence of low-dose Cd exposure alone. Rather, the contributions of environmental Cd, iron deficiency for any cause and iron infusion cannot be separated, and previous studies have considered only iron infusion as the sole cause. Our working hypothesis to explain the involvement of Cd through iron deficiency is presented in Figure 2.

Osteoblasts can easily uptake circulating Cd when it is bound to transferrin [88] and transferrin receptor 1 (TfR1) is expressed on the osteoblast surface [89]. The expression of TfR1 is upregulated by IDA due to the accumulation of hypoxia-inducible factors (HIFs) [90]. Following its entry into osteoblasts, Cd promotes the secretion of iFGF23, which then reduces tubular reabsorption of phosphate, as demonstrated previously [41,42]. IDA also increased the abundance of TfR1 on the cell surface [91,92], resulting in an enhanced uptake of Cd, since, as discussed, Cd is bound to transferrin [92]. In effect, osteoblasts could take up and accumulate Cd for a prolonged period, and Cd promotes mitochondrial production of excessive ROS (Section 4), leading to some extent cell damage. When there is suddenly a significant amount of iron in circulation, osteoblasts take it up as well. Results of an in vivo study demonstrated that Cd toxicity is potentiated in the presence of iron [93]. It would not be possible to ascertain whether those individuals would develop osteomalacia after iron infusion if they were minimally exposed to Cd. However, this could be shown if chelation therapy were applied before iron infusion.

### 3.2. Fracture Risk

Anemia and iron deficiency have been linked to the risk of fractures and postoperative complications after hip fracture [94,95,96,97]. Lee et al. analyzed fracture risk in a large retrospective study that recruited over 70,000 individuals, of which 10,568 (15.1%) had anemia (not classified) [97]. They observed that both men and women with anemia had an increased risk of sustaining vertebral and femoral fractures; moreover, a negative correlation was found between hemoglobin levels and fracture risk for both sites examined. A higher fracture risk was independently associated with current smoking status in another study [97].

In a study from Sweden, an increased risk of incident osteoporosis-related fractures was observed in those who never smoked; the risk of fracture rose 58% per 1 μg/L increase in blood Cd. This result was obtained after adjustment for age, sex, BMI, physical activity, and fiber consumption [98]. A blood Cd of as little as 0.31 µg/L appeared to be sufficient to increase fracture risk by 21%, compared to blood Cd < 0.15 μg/L [98].

A Swedish prospective study conducted on 1005 men (66 with anemia) who participated in the Osteoporotic Fractures in Men (MrOS) study evaluated whether anemia (not classified) could be associated with an increased fracture risk [99]. The median follow-up time in that study was 10.1 years, during which 346 participants sustained fractures. The hazard ratio for any fracture type was almost doubled, even after adjusting for age and hip bone mineral density (BMD). Of note, individuals with anemia had higher circulating levels of intact FGF23 (iFGF23) independent of age, erythropoietin levels and eGFR) [99].

Similarly, Valderrábano et al. examined the association between anemia and fracture risk in 3632 aged men; 249 had anemia [100]. Their results showed that the presence of anemia increased the risk of any fracture type by 67%, while the risk for non-vertebral fractures was even slightly more pronounced [100].

A study from Norway examined whether anemia could predict non-vertebral fractures [101]. This study included 5286 individuals (2511 men and 2775 women), aged 55 to 74 years. As in the study on Swedish men [100], Jørgensen and colleagues found that men with anemia had a twofold higher risk of non-vertebral fractures compared with men with desirable hemoglobin levels. However, after adjusting for various variables such as lipid profiles, forearm BMD, hand grip strength, BMI, smoking status and creatinine levels, the significant effect of anemia on fracture risk was lost for women but remained significant for men [101].

Teng and colleagues conducted a meta-analysis of data from seven original articles [95]. They found that the pooled relative risk for any fracture type in individuals with anemia was 1.26. After stratifying the risk by location, they reported a higher fracture risk for hip fractures than for vertebral fractures in individuals with anemia. Additionally, men had a greater fracture risk than women, although the presence of anemia led to increased fracture risk in both sexes and for both examined sites [95]. Results from another study from the US, which included 160,080 women, indicated an increased risk of spine, hip and all-type fractures, with hip fracture risk being particularly high in individuals with anemia (hazard ratio 1.81) [102].

In a meta-analysis of 18 studies published between 2007 and 2024 (*n* = 861,540), Tear et al. found that anemia was associated with a 1.62-fold increase in the prevalence of osteoporosis (95% CI: 1.33–1.98) and a 1.51-fold increase in the prevalence of osteoporotic fractures (95% CI: 1.26–1.81) [103].

### 3.3. IDA and Other Potential Contributors

Most studies have shown that IDA and iron deficiency have direct effects on bone quality [76,77]. Pioneering work in the field of IDA-induced bone alterations revealed that rats fed with an iron-deficient diet had lower bone mineral density in the femur and spine and worse mechanical properties compared with rats with adequate iron intake [77].

In an in vivo study where rats were fed a low-iron diet for 5 weeks, the low-iron-intake group had lower whole-body and femur DXA scores and deteriorated lumbar microarchitecture. This deterioration was reflected in lower bone volume fraction (BV/TV), trabecular thickness (Tb.Th) and trabecular number (Tb.N), along with a higher structure model index and trabecular separation. In another study using Wistar rats fed with an iron-deficient diet, Diaz-Castro et al. observed decreasing levels of procollagen type I N-terminal propeptide, coupled with rising serum parathyroid hormone, indicative of bone resorption [76].

Osteomalacia due to vitamin D deficiency is characterized by altered mineralization of newly formed osteoid. Chwalba et al. divided 140 children into two groups based on whether their blood Cd levels were above or below the median (0.27 µg/L) and reported that children with blood Cd above the median had 23% lower serum vitamin D levels [104]. There is some evidence that Cd could disrupt the final hydroxylation reaction of provitamin D in the kidneys [105,106].

In summary, IDA unequivocally impacts bone quality. This means that IDA-induced adverse bone effects may also contribute to fractures, especially in cases where osteomalacia follows an iron infusion treatment. Vilaca et al. reported that 22 individuals sustained fractures [71]. Since IDA causes bone alteration and it is speculated that iron infusion can also damage bones, it is difficult to distinguish whether the fractures occurred due to IDA, iron infusion, or a combination of these. Moreover, IDA can raise circulating iFGF23 concentrations, as confirmed in in vivo [107,108,109] and human [110,111] studies. Serum iFGF23 concentrations inversely correlate with serum Fe levels [111]. Upregulation of the FGF23 gene by anemia, iron deficiency and inflammation, through HIF1α and erythropoietin, is being increasingly reported.

## 4. Bone Toxicity Mechanism of Cd

In this section, we highlight another breakthrough study that provides cutting-edge knowledge on transporters responsible for the uptake and accumulation of Cd by osteoblasts. Results from population-based investigations on effects of Cd on bones are summarized, together with empirical studies supporting epidemiological/clinical data provided in Section 3. Additionally, a two-hit hypothesis is presented to explain the molecular basis of bone toxicity due to Cd.

### 4.1. Uptake and Accumulation of Cd by Osteoblasts

In another breakthrough study, using rat osteoblast UMR-106 cells, Fujishiro et al. first identified transport proteins and channels that mediate Cd uptake by osteoblasts, which included ZIP8, ZIP14, DMT1 and two voltage-gated Ca^2+^ channels, TRPV6 and TRPM7. Using siRNA transfection that suppressed the expression of ZIP8, ZIP14, DMT1, CaV1.3 (a component of L-type Ca^2+^ channels), TRPV6, or TRPM7 resulted in 15–35% reductions in Cd uptake by osteoblasts [112]. Hence, Cd can enter osteoblasts through transport proteins for iron, zinc, manganese and calcium. We speculate that the expression of DMT1, FPN1 and TrfR in osteoblasts is all upregulated as an adaptive response to iron deficiency.

### 4.2. Cd Effects on Bones: Human Population Data

Li et al. conducted a meta-analysis of 17 studies, where they observed a statistically significant association between risk of osteoporosis and urinary Cd, but not blood Cd [113]. Using a continuous model estimating BMD and osteoporosis risk, Pouillot et al. linked urinary Cd to osteoporosis in 16% of the U.S. population, aged 50–79 years [32]. Apparently, urinary Cd is a more reliable predictor of osteoporosis than blood Cd because it reflects lifetime exposure or body burden (Section 2.3). Bone effects of Cd observed in the general populations of various countries are listed in Table 1.

### 4.3. Cd Effects on Bones: Experimental Data

Using in vitro experiments, Cd-intoxicated osteoblasts were less viable, and they secreted less alkaline phosphatase [120]. Another study provided evidence that Cd stimulated bone resorption by upregulating RANK expression [121]. Interestingly, Wan et al. demonstrated that Cd per se may affect bone mineralization [122]. Namely, they investigated the effects of Cd on human bone marrow mesenchymal stem cells in vitro and obtained that Cd-exposed cells at the doses of 2.5 and 5 μM CdCl_2_ lacked intracellular calcification nodules in terms of quantity and volume detected by alizarin red staining [122]. In the second part of their study, Wan et al. showed a negative relationship between bone morphogenetic protein 4 and urinary Cd levels [122]. Bone morphogenetic protein 4 has a significant role in bone mineralization [91,123].

Liu et al. investigated mechanisms by which Cd induced osteoblast cell death [118], where they employed osteoblasts derived from Sprague-Dawley rat fetuses treated with 0, 1, 2 and 5 μM Cd. They observed changes in osteoblast nucleus morphology and the upregulation of Bax, as well as the downregulation of Bcl-2 (which has antiapoptotic activity) [124]. In another in vitro study utilizing MC-3T3-E1 cells, exposure to CdCl_2_ at 0–20 μM decreased cell viability and promoted osteoblast apoptosis, which resulted from a decreased Bcl-2 level and accumulation of Bax mRNA and protein, and interfered with osteoblast formation by decreasing the expression of RANKL [125].

In addition to the induction of FGF23 expression in the osteoblast-like cells [40], a series of experimental studies have ascertained Cd’s effects on osteoclasts and osteoblasts via multiple mechanisms, including RANKL-RANK axis [124,125]. Ran et al. demonstrated that the SIRT1/PGC-1α/P53Lys382 signaling pathway could mediate osteoporosis due to Cd, while noting that bones from patients with osteoporosis had an elevated Cd level [126].

In summary, Cd promotes bone resorption by causing the premature death (ferroptosis) of osteoblasts, while increasing the formation of osteoclasts. These findings are in line with current views on the significance of ferroptosis in normal bone health [127,128] and pathogenesis of osteoporosis associated with chronic Cd exposure [126,129,130].

### 4.4. Hypothetical Two-Hit Mechanism of the Cytotoxicity of Cd

The potential contribution of iron-dependent cell death (ferroptosis) in pathogenesis of osteomalacia in itai-itai disease patients was investigated by Noda et al., who examined 23 autopsy cases of itai-itai disease and 18 cases of sudden death as controls [131]. Using histochemical staining and x-ray microanalysis, iron was found in approximately half the mineralization fronts, and the bone content of Cd was 4.5 times higher in patients with itai-itai disease compared to control subjects [131]. The presence of iron at mineralization fronts was also found in rats and monkeys treated with Cd [39,132].

**Figure 3:**
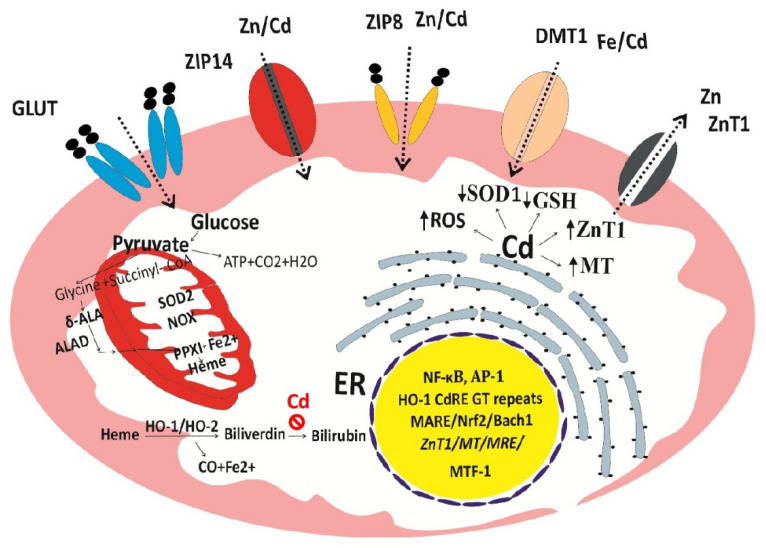
The two-hit hypothesis of the cytotoxicity of Cd. Through *CdRE* and *ARE*, Cd induces a massive increase in HO-1 activity with a release of Fe^2+^ from heme without a concomitant increase in bilirubin synthesis, thereby depriving cells of the ability to defend against lipid peroxidation due to excessive ROS. Cd induces expression of ZnT1, a zinc-specific efflux transporter that can lead to intracellular zinc depletion.

Given the unique and universal role of heme oxygenase-1 (HO-1) and cellular stress response and defense against oxidative damage [133,134], we hypothesize that, through excessive ROS, damage to mitochondria and massive HO-1 induction, Cd causes a release of iron from heme, ultimately leading to cell death through ferroptosis, an iron-dependent form of regulated cell death triggered by lipid peroxidation [135] (Figure 3).

It is important to note, firstly, that Cd at very low concentrations can cause a massive increase in HO-1 protein and its catalytic activity because it activates the HO-1 gene through the cadmium response element (*CdRE*), in addition to the antioxidant response element (*ARE*) located in the promoter region of the HO-1 gene [136,137,138]. Secondly, the increase in HO-1 enzyme activity by Cd is not coupled with bilirubin synthesis [139,140]. In effect, Cd increases intracellular levels of Fe^2+^, while depriving cells of the ability to protect themselves against lipid peroxidation; consequently, ferroptosis ensues. The induced expression of ZnT1 by Cd can lead to cellular zinc depletion because ZnT1 has an absolute specificity for zinc, while FPN1 provides an exit route for Fe^2+^, zinc and cobalt but not Cd.

### 4.5. Zinc Mitigates the Cytotoxicity of Cd

Early experimental studies show that zinc reduced osteoclast activities and increased the number of osteoblasts [141,142]. Conversely, zinc deficiency resulted in oxidative stress and upregulation of RANKL in rat bones [143].

Effects of zinc on the mitigation of bone toxicity of Cd have been demonstrated using BALB/c female mice fed with Cd at 5 and 50 mg/L of drinking water alone or with Zn (30 mg/L); Qin et al. observed a reduction in Cd accumulation in femur bones in groups given Cd plus zinc for 12 months [144]. These results can be expected considering current knowledge on ZIP8 and ZIP14, which mediate Cd and Zn uptake by osteoblasts [112]. Reduced Cd accumulation in bones caused by zinc could explain the differences in severity of mineral loss and femoral trabecular bone pathologies in mice treated with Cd only versus Cd plus zinc groups [144]. As expected, upregulation of MT and MTF-1 was also observed in the Cd plus zinc group, given that zinc is a known inducer of MT and MTF-1 and that zinc is required to achieve maximal induction of MT [145,146]. Sequestration of Cd by nascent MT generation in response to zinc could thus explain a reported reduction in bone toxicity of Cd in mice treated with Cd plus zinc [144].

Using rats fed with Cd (0, 5, 50 mg/L) plus zinc (0, 30, 60 mg/L) in drinking water for 12 months, Bijowski et al. reported a fall in zinc concentrations in mandibular bone tissues due to Cd, which could be reversed by zinc [147]. The authors found that Cd at 5 and 50 mg/L decreased mandibular bone zinc concentrations by 28 and 27%, respectively. In comparison, the mandibular bone zinc concentrations in groups treated with Cd (5 mg/L plus zinc (30 mg/L) and Cd (5 mg/L) plus zinc (60 mg/L) rose 37 and 39%, respectively. Mandibular bone zinc concentrations in groups treated with Cd (50 mg) plus zinc (30 mg/L) and Cd (50 mg) plus zinc (60 mg/L) rose 33% and 31%, respectively.

The above findings underscore the ability of zinc to mitigate the toxicity of Cd by preventing zinc loss and a reduction in intracellular zinc concentrations [147]. Notably, increasing zinc from 30 to 60 mg/L did not result in a further increase in mandibular bone zinc levels, nor did it reduce any Cd accumulation. These results suggest that zinc may be extruded from cells by ZnT1, which is induced by zinc, thereby preventing toxicity from zinc overload. Due to its high specificity, only zinc is excreted, while bone Cd remains unchanged.

## 5. Conclusions

A combined effect of iron deficiency and Cd bone toxicity may partially explain bone complications of intravenous iron supplementation therapy. The synthesis and cleavage of bone-derived FGF23, forming iFGF23 and Cter-FGF23 peptide fragments, are affected by IDA and iron deficiency. The Cter-FGF23 fragment has been linked to iron homeostasis independent of iFGF23’s role in renal phosphate reabsorption. In experimental studies, Cd increased FGF23 expression in osteoblast-like cells and suppressed FGF23 cleavage, leading to a rise in serum FGF23, which, in turn, mediated an effect of Cd on tubular phosphate reabsorption. Moreover, Cd may interfere with vitamin D metabolism and bone mineralization.

While a rising Cd body burden might be a contributing factor, IDA may, in part, account for an increased prevalence of fractures due to Cd because IDA increases Cd absorption and accumulation in bones; bone tissues from patients with osteoporosis had higher Cd levels compared to controls. However, iron deficiency and Cd could raise plasma iFGF23 levels independently. Current evidence suggests a distinct role for the Cter-FGF23 fragment in the regulation of iron homeostasis, while suppressing erythropoietin synthesis in the kidneys via FGF23 contributes further to anemia.

Evidence that environmental Cd increases the prevalence of iron deficiency and IDA in the general population calls for an effort to address a range of environmental exposures in future iron supplementation programs. The ability of Cd to reduce iron absorption, resulting in iron deficiency in Cd toxicity targets, has recently been demonstrated using long-term feeding studies. Previous short-term dosing experiments failed to reveal such effects of Cd on iron absorption, which lead to iron deficiency if exposure to the metal continues, a likely scenario because Cd exposure occurs through a normal diet.

Through *CdRE* and *MARE*/*Nrf*_2_, Cd induces a massive increase in HO-1 enzyme activity, resulting in a release of iron (Fe^2+^) from heme. However, through a mechanism that remains unknown, the upregulation of HO-1 in response to Cd is not coupled with the generation of bilirubin, a potent lipid peroxidation chain breaker; consequently, extensive cellular oxidative damage and cell death (ferroptosis) may ensue.

At very low concentrations, Cd induces the expression of ZnT1, a highly specific zinc efflux transporter; consequently, zinc deficiency is inevitable because zinc is extruded from cells by ZnT1 while Cd is retained within cells. Like bilirubin synthesis, which is impaired by Cd, cellular zinc deficiency is a universal cytotoxic mechanism of Cd.

Avoidance of foods containing high levels of Cd and smoking cessation are pivotal, as is the maintenance of optimal body contents of iron and zinc. Dietary antioxidants provide a complementary preventive measure.

## Figures and Tables

**Table 1:** Environmental Cd exposure levels associated with adverse effects on bones.

Study Population	Cd Exposure Levels and Bone Effects	Reference
Sweden 936 men, aged 70–81 years at baseline (2002 to 2004).	OR for nonvertebral osteoporosis fractures rose 30–40% for every 1 μg Cd/g creatinine increment. OR for osteoporosis-related fractures was increased by Cd exposure through a normal diet and tobacco smoking.	Wallin et al. 2016 [114]
Sweden 886 men, aged 70–81 years at baseline (2002–2004). Baseline mean urinary Cd was 0.25 μg/g creatinine.	Smoking was associated with an elevated risk of all fractures and major osteoporosis fractures. One-half of the total effects of smoking on risk of nonvertebral osteoporosis fractures and hip fractures were related to Cd exposure from smoking.	Li et al. 2020 [115]
Thailand Three-year follow-up (2019–2022). 393 (40 men, 353 women), aged ≥35 years.	A decrease in BMD was observed in the group with urinary Cd levels < 2 μg/g creatinine (*p* = 0.001). Baseline mean urinary Cd fell from 0.392 to 0.384 μg/g creatinine (*p* = 0.004) over three years.	La-Up et al. 2025 [116]
Korea 243 (122 men, 121 women), mean age 57.6 in men and 55.9 years in women. 103 (42.4%) had blood Cd levels > 1.0 μg/L.	In all subjects, blood Cd levels > 1.0 μg/L were associated with a 2.67-fold increase in risk of osteoporosis, adjusting for potential confounders. In women, blood Cd levels 0.5–1.0 and >1 μg/L were, respectively, associated with 3.8-fold and 4.2-fold increases in risk of osteoporosis.	Chung et al. 2022 [117]
U.S. NHANES, 2013–2014 and 2017–2018 cycles 1290 (637 men, 653 women), mean age (range) 59 (48–67) years. 79 (6.12%) were diagnosed with osteoporosis.	Respective risk of osteoporosis rose 3.3-fold and 4.5-fold in those with blood Cd quartiles 3 and 4, compared with the bottom quartile of blood Cd, adjusting for covariates including Pb, Hg, Se and Mn. Mediation analysis estimated that inflammation and oxidative stress accounted for 10 and 4% of the total effect of Cd on risk of osteoporosis, respectively.	Li et al. 2025 [118]
Brazil 380 women, aged 50–70 years. 73 (19.2%) were diagnosed with osteoporosis based on BMD T-scores at the lumbar spine, femoral neck and total hip.	Osteoporosis was associated with urinary Cd, Mn, Pb and Sb excretion rates. Mean values for urinary Cd and urinary Sb were 0.30 and 0.19 µg/g creatinine, respectively. Risk for osteoporosis was increased 1.5-fold by Cd and 2.1-fold by Sb.	Kunioka et al. 2025 [119].

OR, odds ratio; BMD, bone mineral density; NHANES, National Health and Nutrition Examination Survey; Pb, lead; Hg, mercury; Se, selenium; Mn, manganese; Sb, antimony.

## Data Availability

No new data were created or analyzed in this study.

## References

[1] Lopez A., Cacoub P., Macdougall I.C., Peyrin-Biroulet L. (2016). Iron deficiency anaemia. Lancet.

[2] Cappellini M.D., Musallam K.M., Taher A.T. (2020). Iron deficiency anaemia revisited. J. Intern. Med..

[3] Kumar A., Sharma E., Marley A., Samaan M.A., Brookes M.J. (2022). Iron deficiency anaemia: Pathophysiology, assessment, practical management. BMJ Open Gastroenterol..

[4] Auerbach M., DeLoughery T.G., Tirnauer J.S. (2025). Iron Deficiency in Adults: A Review. JAMA.

[5] Li X., Finberg K.E. (2025). Iron Deficiency Anemia. Adv. Exp. Med. Biol..

[6] Ueda N., Takasawa K. (2018). Impact of Inflammation on Ferritin, Hepcidin and the Management of Iron Deficiency Anemia in Chronic Kidney Disease. Nutrients.

[7] Cappellini M.D., Motta I. (2015). Anemia in Clinical Practice-Definition and Classification: Does Hemoglobin Change with Aging?. Semin. Hematol..

[8] GBD 2021 Anaemia Collaborators (2023). Prevalence, years lived with disability, and trends in anaemia burden by severity and cause, 1990–2021: Findings from the Global Burden of Disease Study 2021. Lancet Haematol..

[9] Safiri S., Kolahi A.A., Noori M., Nejadghaderi S.A., Karamzad N., Bragazzi N.L., Sullman M.J.M., Abdollahi M., Collins G.S., Kaufman J.S. (2021). Burden of anemia and its underlying causes in 204 countries and territories, 1990–2019: Results from the Global Burden of Disease Study 2019. J. Hematol. Oncol..

[10] Saini M., Trehan K., Thakur S., Modi A., Jain S.K. (2025). Advances in Iron Deficiency Anaemia Management: Exploring Novel Drug Delivery Systems and Future Perspectives. Curr. Drug Deliv..

[11] Kolarš B., Mijatović Jovin V., Živanović N., Minaković I., Gvozdenović N., Dickov Kokeza I., Lesjak M. (2025). Iron Deficiency and Iron Deficiency Anemia: A Comprehensive Overview of Established and Emerging Concepts. Pharmaceuticals.

[12] Batchelor E.K., Kapitsinou P., Pergola P.E., Kovesdy C.P., Jalal D.I. (2020). Iron Deficiency in Chronic Kidney Disease: Updates on Pathophysiology, Diagnosis, and Treatment. J. Am. Soc. Nephrol..

[13] Moum B., Lindgren S. (2025). Iron Deficiency and Iron Deficiency Anemia in Chronic Disease-Common, Important, and Treatable. J. Clin. Med..

[14] Pan M.-L., Chen L.-R., Tsao H.-M., Chen K.-H. (2017). Iron Deficiency Anemia as a Risk Factor for Osteoporosis in Taiwan: A Nationwide Population-Based Study. Nutrients.

[15] Tari E., Vörhendi N., Kiss S., Teutsch B., Váradi A., Sisák K., Alizadeh H., Hegyi P., Erőss B. (2023). Anaemia Is Associated with an Increased Risk of Fractures, a Systematic Review, and Meta-Analysis. Gerontology.

[16] Chuang M.H., Chuang T.L., Koo M., Wang Y.F. (2019). Low Hemoglobin Is Associated with Low Bone Mineral Density and High Risk of Bone Fracture in Male Adults: A Retrospective Medical Record Review Study. Am. J. Men’s Health.

[17] Lichtler R., Cowley M. (2025). Environmental Contaminants, Iron Deficiency, and Iron-Deficiency Anemia: A Review of the Literature. Scientifica.

[18] Steinbicker A.U., Pantopoulos K. (2025). Oral and Intravenous Iron Therapy. Adv. Exp. Med. Biol..

[19] Pozzessere S. (2025). Iron-Induced Hypophosphatemia: A Review of Pathophysiology, Drug Safety, and Pharmacogenomic Perspectives. J. Hematol..

[20] Strubbe M., David K., Peene B., Eeckhout B., Van der Schueren B., Decallonne B., Vangoitsenhoven R., Vanderschueren D., Antonio L. (2025). No longer to be ignored: Hypophosphatemia following intravenous iron administration. Rev. Endocr. Metab. Disord..

[21] Courbon G., David V. (2024). Fibroblast growth factor 23 is pumping iron: C-terminal-fibroblast growth factor 23 cleaved peptide and its function in iron metabolism. Curr. Opin. Nephrol. Hypertens..

[22] Cifuentes A., Laskar-Marchesseau Z., Courbon G. (2025). FGF23: A player not only in bone diseases. Jt. Bone Spine.

[23] Cantoral A., Collado-López S., Betanzos-Robledo L., Lamadrid-Figueroa H., García-Martínez B.A., Ríos C., Díaz-Ruiz A., Mariscal-Moreno R.M., Téllez-Rojo M.M. (2024). Dietary Risk Assessment of Cadmium Exposure Through Commonly Consumed Foodstuffs in Mexico. Foods.

[24] Zhu H., Tang X., Gu C., Chen R., Liu Y., Chu H., Zhang Z. (2024). Assessment of human exposure to cadmium and its nephrotoxicity in the Chinese population. Sci. Total Environ..

[25] Kolbaum A.E., Jung C., Jaeger A., Libuda L., Lindtner O. (2024). Assessment of long-term dietary cadmium exposure in children in Germany: Does consideration of data from total diet studies reduce uncertainties from food monitoring programmes?. Food Chem. Toxicol..

[26] Hill D.T., Jandev V., Petroni M., Atallah-Yunes N., Bendinskas K., Brann L.S., Heffernan K., Larsen D.A., MacKenzie J.A., Palmer C.D. (2023). Airborne levels of cadmium are correlated with urinary cadmium concentrations among young children living in the New York state city of Syracuse, USA. Environ. Res..

[27] Almerud P., Zamaratskaia G., Lindroos A.K., Bjermo H., Andersson E.M., Lundh T., Ankarberg E.H., Lignell S. (2021). Cadmium, total mercury, and lead in blood and associations with diet, sociodemographic factors, and smoking in Swedish adolescents. Environ. Res..

[28] Fagerberg B., Barregard L. (2021). Review of cadmium exposure and smoking-independent effects on atherosclerotic cardiovascular disease in the general population. J. Intern. Med..

[29] Kim J., Song H., Lee J., Kim Y.J., Chung H.S., Yu J.M., Jang G., Park R., Chung W., Oh C.-M. (2023). Smoking and passive smoking increases mortality through mediation effect of cadmium exposure in the United States. Sci. Rep..

[30] Satarug S. (2024). Is Chronic Kidney Disease Due to Cadmium Exposure Inevitable and Can It Be Reversed?. Biomedicines.

[31] Kunioka C.T., Manso M.C., Carvalho M. (2022). Association between Environmental Cadmium Exposure and Osteoporosis Risk in Postmenopausal Women: A Systematic Review and Meta-Analysis. Int. J. Environ. Res. Public Health.

[32] Pouillot R., Santillana Farakos S., Van Doren J.M. (2022). Modeling the risk of low bone mass and osteoporosis as a function of urinary cadmium in U.S adults aged 50–79 years. Environ. Res..

[33] Nogawa K., Sakurai M., Ishizaki M., Kido T., Nakagawa H., Suwazono Y. (2017). Threshold limit values of the cadmium concentration in rice in the development of itai-itai disease using benchmark dose analysis. J. Appl. Toxicol..

[34] Satarug S., Phelps K.R., Bagchi D., Bagchi M. (2020). Cadmium Exposure and Toxicity. Metal Toxicology Handbook, Ch 14.

[35] Kasuya M. (2000). Recent epidemiological studies on itai-itai disease as a chronic cadmium poisoning in Japan. Water Sci. Technol..

[36] Baba H., Tsuneyama K., Kumada T., Aoshima T., Imura J. (2014). Histopathological analysis for osteomalacia and tubulopathy in itai-itai disease. J. Toxicol. Sci..

[37] Aoshima K. (2016). Itai-itai disease: Renal tubular osteomalacia induced by environmental exposure to cadmium—Historical review and perspectives. Soil Sci. Plant Nutr..

[38] Sasaki T., Horiguchi H., Matsukawa T., Kobayashi M., Omori Y., Oguma E., Komatsuda A. (2024). A suspected case of “itai-itai disease” in a cadmium-polluted area in Akita prefecture, Japan. Environ. Health Prev. Med..

[39] Kurata Y., Katsuta O., Doi T., Kawasuso T., Hiratsuka H., Tsuchitani M., Umemura T. (2014). Chronic cadmium treatment induces tubular nephropathy and osteomalacic osteopenia in ovariectomized cynomolgus monkeys. Vet. Pathol..

[40] Kido S., Fujihara M., Nomura K., Sasaki S., Mukai R., Ohnishi R., Kaneko I., Segawa H., Tatsumi S., Izumi H. (2014). Molecular mechanisms of cadmium-induced fibroblast growth factor 23 upregulation in osteoblast-like cells. Toxicol. Sci..

[41] Aranami F., Segawa H., Furutani J., Kuwahara S., Tominaga R., Hanabusa E., Tatsumi S., Kido S., Ito M., Miyamoto K. (2010). Fibroblast growth factor 23 mediates the phosphaturic actions of cadmium. J. Med. Investig..

[42] Nishito Y., Kambe T. (2018). Absorption mechanisms of iron, copper, and zinc: An overview. J. Nutr. Sci. Vitaminol..

[43] Kondaiah P., Yaduvanshi P.S., Sharp P.A., Pullakhandam R. (2019). Iron and zinc homeostasis and interactions: Does enteric zinc excretion cross-talk with intestinal iron absorption?. Nutrients.

[44] Okazaki Y. (2024). Iron from the gut: The role of divalent metal transporter 1. J. Clin. Biochem. Nutr..

[45] Kovacs G., Danko T., Bergeron M.J., Balazs B., Suzuki Y., Zsembery A., Hediger M.A. (2011). Heavy metal cations permeate the TRPV6 epithelial cation channel. Cell Calcium.

[46] Kovacs G., Montalbetti N., Franz M.C., Graeter S., Simonin A., Hediger M.A. (2013). Human TRPV5 and TRPV6: Key players in cadmium and zinc toxicity. Cell Calcium.

[47] Aydemir T.B., Cousins R.J. (2018). The multiple faces of the metal transporter ZIP14 (SLC39A14). J. Nutr..

[48] Schneider S.N., Liu Z., Wang B., Miller M.L., Afton S.E., Soleimani M., Nebert D.W. (2014). Oral cadmium in mice carrying 5 versus 2 copies of the Slc39a8 gene: Comparison of uptake, distribution, metal content, and toxicity. Int. J. Toxicol..

[49] Nebert D.W. (2015). Comparing gene expression during cadmium uptake and distribution: Untreated versus oral Cd-treated wild-type and ZIP14 knockout mice. Toxicol. Sci..

[50] Park J.D., Cherrington N.J., Klaassen C.D. (2002). Intestinal absorption of cadmium is associated with divalent metal transporter 1 in rats. Toxicol. Sci..

[51] Fujita Y., el Belbasi H.I., Min K.S., Onosaka S., Okada Y., Matsumoto Y., Mutoh N., Tanaka K. (1993). Fate of cadmium bound to phytochelatin in rats. Res. Commun. Chem. Pathol. Pharmacol..

[52] Langelueddecke C., Roussa E., Fenton R.A., Thévenod F. (2013). Expression and function of the lipocalin-2 (24p3/NGAL) receptor in rodent and human intestinal epithelia. PLoS ONE.

[53] Langelueddecke C., Lee W.K., Thévenod F. (2014). Differential transcytosis and toxicity of the hNGAL receptor ligands cadmium-metallothionein and cadmium-phytochelatin in colon-like Caco-2 cells: Implications for in vivo cadmium toxicity. Toxicol. Lett..

[54] Mitchell C.J., Shawki A., Ganz T., Nemeth E., Mackenzie B. (2014). Functional properties of human ferroportin, a cellular iron exporter reactive also with cobalt and zinc. Am. J. Physiol. Cell Physiol..

[55] Frazer D.M., Anderson G.J., Collins J.F. (2025). Dietary Iron Absorption: Biochemical and Nutritional Aspects. Adv. Exp. Med. Biol..

[56] Hoch E., Lin W., Chai J., Hershfinkel M., Fu D., Sekler I. (2012). Histidine pairing at the metal transport site of mammalian ZnT transporters controls Zn^2+^ over Cd^2+^ selectivity. Proc. Natl. Acad. Sci. USA.

[57] Järup L., Rogenfelt A., Elinder C.G., Nogawa K., Kjellström T. (1983). Biological half-time of cadmium in the blood of workers after cessation of exposure. Scand. J. Work Environ. Health.

[58] Elinder C.G., Lind B., Kjellström T., Linnman L., Friberg L. (1976). Cadmium in kidney cortex, liver, and pancreas from Swedish autopsies. Estimation of biological half time in kidney cortex, considering calorie intake and smoking habits. Arch. Environ. Health.

[59] Suwazono Y., Kido T., Nakagawa H., Nishijo M., Honda R., Kobayashi E., Dochi M., Nogawa K. (2009). Biological half-life of cadmium in the urine of inhabitants after cessation of cadmium exposure. Biomarkers.

[60] Ishizaki M., Suwazono Y., Kido T., Nishijo M., Honda R., Kobayashi E., Nogawa K., Nakagawa H. (2015). Estimation of biological half-life of urinary cadmium in inhabitants after cessation of environmental cadmium pollution using a mixed linear model. Food Addit. Contam. Part A Chem. Anal. Control Expo. Risk Assess..

[61] Satarug S., Vesey D.A., Ruangyuttikarn W., Nishijo M., Gobe G.C., Phelps K.R. (2019). The Source and Pathophysiologic Significance of Excreted Cadmium. Toxics.

[62] Sun H., Wang D., Zhou Z., Ding Z., Chen X., Xu Y., Huang L., Tang D. (2016). Association of cadmium in urine and blood with age in a general population with low environmental exposure. Chemosphere.

[63] Orlowski C., Piotrowski J.K., Subdys J.K., Gross A. (1998). Urinary cadmium as indicator of renal cadmium in humans: An autopsy study. Hum. Exp. Toxicol..

[64] Satarug S., Baker J.R., Reilly P.E., Moore M.R., Williams D.J. (2002). Cadmium levels in the lung, liver, kidney cortex, and urine samples from Australians without occupational exposure to metals. Arch. Environ. Health.

[65] Akerstrom M., Barregard L., Lundh T., Sallsten G. (2013). The relationship between cadmium in kidney and cadmium in urine and blood in an environmentally exposed population. Toxicol. Appl. Pharmacol..

[66] Wallin M., Sallsten G., Lundh T., Barregard L. (2014). Low-level cadmium exposure and effects on kidney function. Occup. Environ. Med..

[67] Tokumoto M., Lee J.-Y., Fujiwara Y., Satoh M. (2023). Long-Term Exposure to Cadmium Causes Hepatic Iron Deficiency through the Suppression of Iron-Transport-Related Gene Expression in the Proximal Duodenum. Toxics.

[68] Zhang K., Long M., Dong W., Li J., Wang X., Liu W., Huang Q., Ping Y., Zou H., Song R. (2024). Cadmium Induces Kidney Iron Deficiency and Chronic Kidney Injury by Interfering with the Iron Metabolism in Rats. Int. J. Mol. Sci..

[69] Giri S., Roy A., Kumar A., Ghosh S., Bhunia A., Patra S. (2025). Cadmium toxicity-related metabolic bone disease: A clinical conundrum of five cases. Osteoporos. Int..

[70] Samões B., Silva B., Martins A., Oliveira D., Rajão Martins F., Fonseca D., Costa L., Bernardes M. (2023). Hypophosphatemic osteomalacia induced by intravenous iron therapy: A case report. Jt. Bone Spine.

[71] Vilaca T., Velmurugan N., Smith C., Abrahamsen B., Eastell R. (2022). Osteomalacia as a Complication of Intravenous Iron Infusion: A Systematic Review of Case Reports. J. Bone Miner. Res..

[72] Bartko J., Roschger P., Zandieh S., Brehm A., Zwerina J., Klaushofer K. (2018). Hypophosphatemia, Severe Bone Pain, Gait Disturbance, and Fatigue Fractures After Iron Substitution in Inflammatory Bowel Disease: A Case Report. J. Bone Miner. Res..

[73] Baban Y.N., Edicheria C.M., Joseph J., Kaur P., Mostafa J.A. (2021). Osteoporosis Complications in Crohn’s Disease Patients: Factors, Pathogenesis, and Treatment Outlines. Cureus.

[74] Siffledeen J.S., Fedorak R.N., Siminoski K., Jen H., Vaudan E., Abraham N., Seinhart H., Greenberg G. (2004). Bones and Crohn’s: Risk factors associated with low bone mineral density in patients with Crohn’s disease. Inflamm. Bowel Dis..

[75] Medeiros D.M., Stoecker B., Plattner A., Jennings D., Haub M. (2004). Iron Deficiency Negatively Affects Vertebrae and Femurs of Rats Independently of Energy Intake and Body Weight. J. Nutr..

[76] Díaz-Castro J., López-Frías M.R., Campos M.S., López-Frías M., Alférez M.J., Nestares T., Ojeda M.L., López-Aliaga I. (2012). Severe nutritional iron-deficiency anaemia has a negative effect on some bone turnover biomarkers in rats. Eur. J. Nutr..

[77] Katsumata S., Katsumata-Tsuboi R., Uehara M., Suzuki K. (2009). Severe iron deficiency decreases both bone formation and bone resorption in rats. J. Nutr..

[78] Katsumata S., Tsuboi R., Uehara M., Suzuki K. (2006). Dietary iron deficiency decreases serum osteocalcin concentration and bone mineral density in rats. Biosci. Biotechnol. Biochem..

[79] Bárány E., Bergdahl I.A., Bratteby L.E., Lundh T., Samuelson G., Skerfving S., Oskarsson A. (2005). Iron status influences trace element levels in human blood and serum. Environ. Res..

[80] Lee B.K., Kim Y. (2012). Iron deficiency is associated with increased levels of blood cadmium in the Korean general population: Analysis of 2008–2009 Korean National Health and Nutrition Examination Survey data. Environ. Res..

[81] Lee B.K., Kim S.H., Kim N.S., Ham J.O., Kim Y. (2014). Iron deficiency increases blood cadmium levels in adolescents surveyed in KNHANES 2010–2011. Biol. Trace Elem. Res.

[82] Cirovic A., Denic A., Clarke B.L., Vassallo R., Cirovic A., Landry G.M. (2022). A hypoxia-driven occurrence of chronic kidney disease and osteoporosis in COPD individuals: New insights into environmental cadmium exposure. Toxicology.

[83] Roy A., Saha T., Sahoo J., Das A. (2024). Hypophosphatemic osteomalacia due to cadmium toxicity in silverware industry: A curious case of aches and pains. J. Fam. Med. Prim. Care.

[84] Balusikova K., Dostalikova-Cimburova M., Tacheci I., Kovar J. (2022). Expression profiles of iron transport molecules along the duodenum. J. Cell. Mol. Med..

[85] Wang C.Y., Jenkitkasemwong S., Duarte S., Sparkman B.K., Shawki A., Mackenzie B., Knutson M.D. (2012). ZIP8 is an iron and zinc transporter whose cell-surface expression is up-regulated by cellular iron loading. J. Biol. Chem..

[86] Ohta H., Ohba K. (2020). Involvement of metal transporters in the intestinal uptake of cadmium. J. Toxicol. Sci..

[87] Klein K., Asaad S., Econs M., Rubin J.E. (2018). Severe FGF23-based hypophosphataemic osteomalacia due to ferric carboxymaltose administration. BMJ Case Rep..

[88] Saljooghi A.S., Fatemi S.J. (2010). Cadmium transport in blood serum. Toxicol. Ind. Health.

[89] Ledesma-Colunga M.G., Weidner H., Vujic Spasic M., Hofbauer L.C., Baschant U., Rauner M. (2021). Shaping the bone through iron and iron-related proteins. Semin. Hematol..

[90] Watts D., Gaete D., Rodriguez D., Hoogewijs D., Rauner M., Sormendi S., Wielockx B. (2020). Hypoxia Pathway Proteins are Master Regulators of Erythropoiesis. Int. J. Mol. Sci..

[91] Luppen C.A., Chandler R.L., Noh T., Mortlock D.P., Frenkel B. (2008). BMP-2 vs. BMP-4 expression and activity in glucocorticoid-arrested MC3T3-E1 osteoblasts: Smad signaling, not alkaline phosphatase activity, predicts rescue of mineralization. Growth Factors.

[92] Cirovic A., Cirovic A. (2023). Letter to the editor for the “relationship between iron deficiency and expression of genes involved in iron metabolism in human myocardium and skeletal muscle”. Int. J. Cardiol..

[93] Cabrera C., Frisk C., Löfström U., Lyngå P., Linde C., Hage C., Persson H., Eriksson M.J., Wallén H., Persson B. (2023). Relationship between iron deficiency and expression of genes involved in iron metabolism in human myocardium and skeletal muscle. Int. J. Cardiol..

[94] Toxqui L., Vaquero M.P. (2015). Chronic iron deficiency as an emerging risk factor for osteoporosis: A hypothesis. Nutrients.

[95] Teng Y., Teng Z., Xu S., Zhang X., Liu J., Yue Q., Zhu Y., Zeng Y. (2020). The Analysis for Anemia Increasing Fracture Risk. Med. Sci. Monit..

[96] Jiang Y., Lin X., Wang Y., Li J., Wang G., Meng Y., Li M., Li Y., Luo Y., Gao Z. (2023). Preoperative Anemia and Risk of In-hospital Postoperative Complications in Patients with Hip Fracture. Clin. Interv. Aging.

[97] Lee E.A., Shin D.W., Yoo J.H., Ko H.Y., Jeong S.M. (2019). Anemia and risk of fractures in older Korean adults: A nationwide population-based study. J. Bone Min. Res..

[98] Wallin M., Andersson E.M., Engström G. (2024). Blood cadmium is associated with increased fracture risk in never-smokers—Results from a case-control study using data from the Malmö Diet and Cancer cohort. Bone.

[99] Kristjansdottir H.L., Mellström D., Johansson P., Karlsson M., Vandenput L., Lorentzon M., Herlitz H., Ohlsson C., Lerner U.H., Lewerin C. (2022). Anemia is associated with increased risk of non-vertebral osteoporotic fractures in elderly men: The MrOS Sweden cohort. Arch. Osteoporos..

[100] Valderrábano R.J., Lee J., Lui L.Y., Hoffman A.R., Cummings S.R., Orwoll E.S., Wu J.Y., Osteoporotic Fractures in Men (MrOS) Study Research Group (2017). Older Men with Anemia Have Increased Fracture Risk Independent of Bone Mineral Density. J. Clin. Endocrinol. Metab..

[101] Jørgensen L., Skjelbakken T., Løchen M.L., Ahmed L., Bjørnerem A., Joakimsen R., Jacobsen B.K. (2010). Anemia and the risk of non-vertebral fractures: The Tromsø Study. Osteoporos. Int..

[102] Chen Z., Thomson C.A., Aickin M., Nicholas J.S., Van Wyck D., Lewis C.E., Cauley J.A., Bassford T., Short list of Women’s Health Initiative Investigators (2010). The relationship between incidence of fractures and anemia in older multiethnic women. J. Am. Geriatr. Soc..

[103] Tear A.A., Tesno F.A., Shofiadeita H., Bunga G.M., Taqiya H.N., Sanditha N.A., Fayyaza A.H., Tambunan S.K.T., Wangsa G.K., Fatimah Q.R. (2026). Association between anaemia and osteoporosis: A systematic review and meta-analysis. Ann. Med..

[104] Chwalba A., Orłowska J., Słota M., Jeziorska M., Filipecka K., Bellanti F., Dobrakowski M., Kasperczyk A., Zalejska-Fiolka J., Kasperczyk S. (2023). Effect of Cadmium on Oxidative Stress Indices and Vitamin D Concentrations in Children. J. Clin. Med..

[105] Moon J. (1994). The role of vitamin D in toxic metal absorption: A review. J. Am. Coll. Nutr..

[106] Schwalfenberg G.K., Genuis S.J. (2015). Vitamin D, Essential Minerals, and Toxic Elements: Exploring Interactions between Nutrients and Toxicants in Clinical Medicine. Sci. World J..

[107] Hanudel M.R., Chua K., Rappaport M., Gabayan V., Valore E., Goltzman D., Ganz T., Nemeth E., Salusky I.B. (2016). Effects of dietary iron intake and chronic kidney disease on fibroblast growth factor 23 metabolism in wild-type and hepcidin knockout mice. Am. J. Physiol. Renal. Physiol..

[108] Clinkenbeard E.L., Farrow E.G., Summers L.J., Cass T.A., Roberts J.L., Bayt C.A., Lahm T., Albrecht M., Allen M.R., Peacock M. (2014). Neonatal iron deficiency causes abnormal phosphate metabolism by elevating FGF23 in normal and ADHR mice. J. Bone Miner. Res..

[109] Li X., Lozovatsky L., Tommasini S.M., Fretz J., Finberg K.E. (2023). Bone marrow sinusoidal endothelial cells are a site of Fgf23 upregulation in a mouse model of iron deficiency anemia. Blood Adv..

[110] Bożentowicz-Wikarek M., Kocełak P., Owczarek A., Olszanecka-Glinianowicz M., Mossakowska M., Skalska A., Więcek A., Chudek J. (2015). Plasma fibroblast growth factor 23 concentration and iron status. Does the relationship exist in the elderly population?. Clin. Biochem..

[111] Lewerin C., Ljunggren Ö., Nilsson-Ehle H., Karlsson M.K., Herlitz H., Lorentzon M., Ohlsson C., Mellström D. (2017). Low serum iron is associated with high serum intact FGF23 in elderly men: The Swedish MrOS study. Bone.

[112] Fujishiro H., Ikeue Y., Himeno S., Sumi D. (2025). Cadmium Uptake into Rat Osteoblast UMR-106 Cells is Mediated via Multiple Pathways. Biol. Trace Elem. Res..

[113] Li D., Lin H., Zhang M., Meng J., Hu L., Yu B. (2021). Urine Cadmium as a Risk Factor for Osteoporosis and Osteopenia: A Meta-Analysis. Front. Med..

[114] Wallin M., Barregard L., Sallsten G., Lundh T., Karlsson M.K., Lorentzon M., Ohlsson C., Mellström D. (2016). Low-Level Cadmium Exposure Is Associated with Decreased Bone Mineral Density and Increased Risk of Incident Fractures in Elderly Men: The MrOS Sweden Study. J. Bone Miner. Res..

[115] Li H., Wallin M., Barregard L., Sallsten G., Lundh T., Ohlsson C., Mellström D., Andersson E.M. (2020). Smoking-Induced Risk of Osteoporosis Is Partly Mediated by Cadmium from Tobacco Smoke: The MrOS Sweden Study. J. Bone Miner. Res..

[116] La-Up A., Saengow U., Umpong T., Buakate P., Oprasertsawat M. (2025). Three-year assessment of cadmium exposure and bone mineral density changes in cadmium-contaminated areas in northwestern Thailand. PLoS ONE.

[117] Chung S.M. (2022). Long-Term Sex-Specific Effects of Cadmium Exposure on Osteoporosis and Bone Density: A 10-Year Community-Based Cohort Study. J. Clin. Med..

[118] Li Z., Jiang R., Qu Q., Mou S., Zhang Z., Zhu W. (2025). Associations of cadmium exposure within heavy metal combinations with osteoporosis risk: An analysis of NHANES data (2013–2014 and 2017–2018). Aging Clin. Exp. Res..

[119] Kunioka C.T., de Oliveira Souza V.C., Rocha B.A., Júnior F.B., Belo L., Manso M.C., Carvalho M. (2025). Association of Urinary Cadmium and Antimony with Osteoporosis Risk in Postmenopausal Brazilian Women: Insights from a 20 Metal(loid) Biomonitoring Study. Toxics.

[120] Al-Ghafari A., Elmorsy E., Fikry E., Alrowaili M., Carter W.G. (2019). The heavy metals lead and cadmium are cytotoxic to human bone osteoblasts via induction of redox stress. PLoS ONE.

[121] Chen X., Zhu G., Gu S., Jin T., Shao C. (2009). Effects of cadmium on osteoblasts and osteoclasts in vitro. Environ. Toxicol. Pharmacol..

[122] Wan Y., Mo L.J., Wu L., Li D.L., Song J., Hu Y.K., Huang H.B., Wei Q.Z., Wang D.P., Qiu J.M. (2023). Bone morphogenetic protein 4 is involved in cadmium-associated bone damage. Toxicol. Sci..

[123] Lademann F., Hofbauer L.C., Rauner M. (2020). The bone morphogenetic protein pathway: The osteoclastic perspective. Front. Cell Dev. Biol..

[124] Liu W., Dai N., Wang Y., Xu C., Zhao H., Xia P., Gu J., Liu X., Bian J., Yuan Y. (2016). Role of autophagy in cadmium-induced apoptosis of primary rat osteoblasts. Sci. Rep..

[125] He T., Shen H., Zhu J., Zhu Y., He Y., Li Z., Lu H. (2019). Geniposide attenuates cadmium-induced oxidative stress injury via Nrf2 signaling in osteoblasts. Mol. Med. Rep..

[126] Ran D., Zhou D., Liu G., Ma Y., Ali W., Yu R., Wang Q., Zhao H., Zhu J., Zou H. (2023). Reactive oxygen species control osteoblast apoptosis through SIRT1/PGC-1α/P53Lys382 signaling, mediating the onset of Cd-induced osteoporosis. J. Agric. Food Chem..

[127] Nan W., Zhou W.M., Zi J.L., Shi Y.Q., Dong Y.B., Song W., Ma Y.C., Zhang H.H. (2025). Ferroptosis and bone metabolic diseases: The dual regulatory role of the Nrf2/HO-1 signaling axis. Front. Cell Dev. Biol..

[128] Yang D., Gong G., Song J., Chen J., Wang S., Li J., Wang G. (2025). Ferroptosis-mediated osteoclast-osteoblast crosstalk: Signaling pathways governing bone remodeling in osteoporosis. J. Orthop. Surg. Res..

[129] Zhang X., Hua Z., Lu Z., Wang B., Wang P., Zhang S., Yang X., Zhang C. (2025). Overview of Ferroptosis in Cadmium Toxicity. Biol. Trace Elem. Res..

[130] Deng P., Li J., Lu Y., Hao R., He M., Li M., Tan M., Gao P., Wang L., Hong H. (2023). Chronic cadmium exposure triggered ferroptosis by perturbing the STEAP3-mediated glutathione redox balance linked to altered metabolomic signatures in humans. Sci. Total Environ..

[131] Noda M., Yasuda M., Kitagawa M. (1991). Iron as a possible aggravating factor for osteopathy in itai-itai disease, a disease associated with chronic cadmium intoxication. J. Bone Miner. Res..

[132] Hiratsuka H., Katsuta O., Toyota N., Tsuchitani M., Akiba T., Marumo F., Umemura T. (1997). Iron deposition at mineralization fronts and osteoid formation following chronic cadmium exposure in ovariectomized rats. Toxicol. Appl. Pharmacol..

[133] Gallio A.E., Marson N.A., Heesom K.J., Lewis P.A., Alibhai D., Dugdale C.A., Herman A., Basran J., Hudson A.J., Raven E.L. (2025). An extended network for regulation of heme homeostasis in cells. Proc. Natl. Acad. Sci. USA.

[134] Simmons S.O., Fan C.Y., Yeoman K., Wakefield J., Ramabhadran R. (2011). NRF2 Oxidative Stress Induced by Heavy Metals is Cell Type Dependent. Curr. Chem. Genom..

[135] Rochette L., Dogon G., Rigal E., Zeller M., Cottin Y., Vergely C. (2023). Lipid Peroxidation and Iron Metabolism: Two Corner Stones in the Homeostasis Control of Ferroptosis. Int. J. Mol. Sci..

[136] Takeda K., Ishizawa S., Sato M., Yoshida T., Shibahara S. (1994). Identification of a cis-acting element that is responsible for cadmium-mediated induction of the human heme oxygenase gene. J. Biol. Chem..

[137] Stewart D., Killeen E., Naquin R., Alam S., Alam J. (2003). Degradation of transcription factor Nrf2 via the ubiquitin-proteasome pathway and stabilization by cadmium. J. Biol. Chem..

[138] Suzuki H., Tashiro S., Sun J., Doi H., Satomi S., Igarashi K. (2003). Cadmium induces nuclear export of Bach1, a transcriptional repressor of heme oxygenase-1 gene. J. Biol. Chem..

[139] Takeda T.A., Mu A., Tai T.T., Kitajima S., Taketani S. (2015). Continuous de novo biosynthesis of haem and its rapid turnover to bilirubin are necessary for cytoprotection against cell damage. Sci. Rep..

[140] Kumagai A., Ando R., Miyatake H., Greimel P., Kobayashi T., Hirabayashi Y., Shimogori T., Miyawaki A. (2013). A bilirubin-inducible fluorescent protein from eel muscle. Cell.

[141] Hadley K.B., Newman S.M., Hunt J.R. (2010). Dietary zinc reduces osteoclast resorption activities and increases markers of osteoblast differentiation, matrix maturation, and mineralization in the long bones of growing rats. J. Nutr. Biochem..

[142] Nagata M., Lönnerdal B. (2011). Role of zinc in cellular zinc trafficking and mineralization in a murine osteoblast-like cell line. J. Nutr. Biochem..

[143] Suzuki T., Katsumata S., Matsuzaki H., Suzuki K. (2016). Dietary zinc deficiency induces oxidative stress and promotes tumor necrosis factor-α- and interleukin-1β-induced RANKL expression in rat bone. J. Clin. Biochem. Nutr..

[144] Qin Y., Fan L., Feng Y., Zhang D., Xiang C. (2026). Zinc antagonizes cadmium-induced osteoporosis in mice by inhibiting Cd (^2+^) deposition, enhancing antioxidant capacity and correcting bone metabolic disorders. Ecotoxicol. Environ. Saf..

[145] Sato M., Mehra R.K., Bremner I. (1984). Measurement of plasma metallothionein-I in the assessment of the zinc status of zinc-deficient and stressed rats. J. Nutr..

[146] Laity J.H., Andrews G.K. (2007). Understanding the mechanisms of zinc-sensing by metal-response element binding transcription factor-1 (MTF-1). Arch. Biochem. Biophys..

[147] Bijowski K., Dąbrowska E., Brzóska M.M., Rogalska J., Orywal K., Dąbrowska Z.N., Borys J. (2025). The Protective Effect of Zinc Supplementation Against Oxidative Stress and Oxidative Modifications of Cellular Macromolecules in the Mandibular Bone of Rats Exposed to Cadmium. Antioxidants.

